# Targeting HDAC3 Suppresses Ferroptosis and Demyelination in White Matter Injury by Restoring PDK4‐Mediated Iron Homeostasis

**DOI:** 10.1111/cns.70471

**Published:** 2025-06-08

**Authors:** Ting Xu, Lisha Ye, Wenfeng Li, Fangming Liu, Tianjiao Wei, Wenhui Gu, Lihua Xu, Mingde Fang, Qianqian Luo, Chuanjie Wu, Guohua Wang

**Affiliations:** ^1^ Department of Neurophysiology and Neuropharmacology, Institute of Special Environmental Medicine and Co‐Innovation Center of Neuroregeneration Nantong University Nantong Jiangsu China; ^2^ Department of Neurology Xuanwu Hospital Capital Medical University Beijing China; ^3^ Beijing Institute of Brain Disorders Capital Medical University Beijing China

**Keywords:** demyelination, ferroptosis, HDAC3, oligodendrocyte, PDK4, RGFP966, white matter injury

## Abstract

**Aim:**

White matter injury (WMI), characterized by white matter degeneration and iron deposition, contributes to neurological dysfunction. Histone deacetylase 3 (HDAC3) is implicated in neurodegenerative processes, yet its role in WMI‐associated ferroptosis remains unclear.

**Methods:**

Clinical assessments in WMI patients revealed correlations between serum iron, α‐synuclein, and antioxidant levels and MRI‐confirmed white matter degeneration. In a cuprizone‐induced demyelination mouse model, white matter integrity, oligodendrocyte dysfunction, iron accumulation, and lipid peroxidation were evaluated through behavioral testing, histological staining, and biochemical analyses. To identify potential molecular targets of HDAC3‐mediated ferroptosis, CUT&Tag sequencing was performed. The involvement of this pathway was further validated in vitro using iron overload assays and in vivo through HDAC3 overexpression via AAV vectors.

**Results:**

In the present study, HDAC3 expression was elevated following demyelination and was suppressed by RGFP966 treatment. Brain MRI findings from clinical patients and histological analyses in CPZ‐treated mice revealed disrupted iron metabolism following white matter injury, likely driven by increased iron deposition and lipid peroxidation in the affected regions. HDAC3 inhibition alleviated oligodendrocyte lineage dysfunction, preserved myelin integrity, and mitigated cognitive and motor deficits induced by demyelination. CUT&Tag sequencing suggested that the therapeutic effects of RGFP966 are mediated through HDAC3‐dependent regulation of ferroptosis. The reliability of these findings was further supported by in vivo validation of ferroptosis‐related gene expression, indicating that the HDAC3–pyruvate dehydrogenase kinase 4 (PDK4) axis plays a critical role in the epigenetic regulation of ferroptosis‐related pathways.

**Conclusion:**

HDAC3 drives ferroptosis in WMI via iron metabolism and lipid peroxidation, highlighting the HDAC3‐PDK4 axis as a therapeutic target.

## Graphical abstract

Targeting the HDAC3‐PDK4 axis mitigated demyelinating‐induced ferroptosis. Iron metabolism disorder is not only a secondary phenomenon of demyelination, but also a vicious cycle through the activation of ferroptosis. Iron overload drives lipid peroxidation, triggering oligodendrocyte death and subsequent iron release, which amplifies oxidative damage. This cascade disrupts myelin repair and accelerates neurological decline.

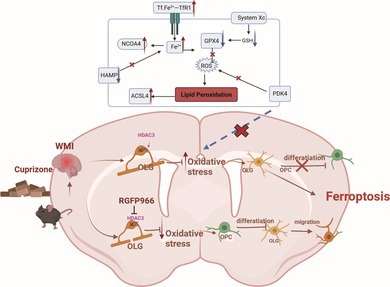

## 1 Introduction

Cognitive dysfunction in neurodegenerative diseases represents a growing public health burden amid global aging. White matter injury (WMI) has been identified as a significant early biomarker of neurodegeneration, correlating with an elevated risk of dementia. This underscores the pressing need for the development of therapeutic strategies that target the underlying mechanisms of WMI [1]. White matter constitutes 50% of brain volume and is responsible for facilitating neural communication via myelinated axons, which are essential for the performance of higher cognitive functions [2, 3]. The pathological degeneration of white matter is characterized by the dysfunction of oligodendrocytes (OLGs), accompanied by demyelination and axonal injury. These processes result in motor deficits, autonomic dysregulation, and cognitive decline, which are core features that are shared across central nervous system (CNS) disorders [4, 5].

Demyelination involves myelin stripping and phagocytic clearance mediated by activated microglia/macrophages through reactive oxygen species (ROS) and nitric oxide, inducing oligodendroglial injury [6, 7]. Combined central–peripheral demyelination manifests as sensory deficits, paralysis, and multifocal CNS lesions [8, 9]. Oligodendrocyte lineage differentiation follows strict temporal regulation: precursor stages express CNPase/GalC, transitioning to APC/CC1 and myelin basic protein (MBP) during maturation [10, 11]. Postmortem analyses in Alzheimer's disease revealed downregulated glycolysis/ketone metabolism genes in oligodendrocytes [12], implicating metabolic dysregulation in functional impairment.

Iron homeostasis critically regulates OLG maturation and myelination [13]. OLGs' iron‐rich phenotype predisposes them to Fenton reaction‐mediated oxidative damage and ferroptosis—an iron‐dependent cell death pathway marked by lipid peroxidation [14, 15, 16]. Neurodegenerative disorders exhibit disease‐specific iron deposition [17]. Chronic inflammation disrupts glutathione metabolism, suppressing glutathione peroxidase 4 (GPX4) activity and unleashing lipid peroxide‐driven ferroptosis cascades [18, 19]. In addition, recurrent white matter ischemia disrupts iron‐containing myelin debris clearance, drives lipid peroxidation product accumulation, and triggers ferroptosis‐mediated neurodegeneration [20]. These breakthrough findings provide a new theoretical framework for elucidation of the molecular mechanism of white matter damage from the perspective of iron metabolism–oxidative stress interaction.

Epigenetic regulation plays an important role in the process of neurodegeneration [21, 22]. Histone acetylation balance, regulated by Histone Acetyltransferases (HATs) and Histone Deacetylases (HDACs), coordinates apoptosis, inflammation, mitochondrial dynamics, and oxidative stress [23]. HDAC dysregulation correlates with synaptic plasticity impairment and cognitive decline [24]. HDAC inhibition enhances synaptic plasticity and attenuates oxidative damage [25, 26]. Histone deacetylase 3 (HDAC3), a class I HDAC, has garnered attention for its dual roles in neurotoxicity and repair [27]. Our prior work identified selective HDAC3 upregulation in the corpus callosum post‐demyelination, reversible by valproic acid (VPA) treatment [28]. Studies show that the HDAC3 inhibitor RGFP966 promotes remyelination via Phosphoinositide 3‐Kinase–Protein Kinase B (PI3K‐AKT) and Mitogen‐Activated Protein Kinase–Extracellular Signal‐Regulated Kinase 1/2 (MAPK‐ERK1/2) activation and mitigates ROS‐mediated apoptosis [29, 30]. In kidney disease, a strategy of inhibiting HDAC3 to preserve GPX4 may be effective in reducing renal ferroptosis and slowing the progression of acute kidney injury to chronic kidney disease [31]. These studies all suggest that Class I HDACs, especially HDAC3, play a role in various neurodegenerative diseases, especially demyelinating diseases, but the specific mechanism of iron homeostasis remains to be studied.

This study integrates clinical Magnetic resonance imaging (MRI) evidence of iron dysregulation in WMI patients with cuprizone (CPZ) mouse model recapitulating iron deposition and myelin loss. Building on prior findings of HDAC3 upregulation post‐injury, we employed in vivo and in vitro approaches to dissect HDAC3's epigenetic role in driving ferroptosis during demyelination.

## 2 Materials and Methods

### 2.1 Clinical Study

#### 2.1.1 Participants

This study enrolled 31 acquired WMI patients from Nantong University Hospital (2021–2022), including 17 males and 14 females. Exclusion criteria: pregnancy/lactation, prior neurointerventions, severe organ dysfunction, intracranial tumors, or pre‐existing neuropsychiatric conditions. Approved by Nantong University Ethics Committee (No. 2020‐39), with informed consent from all participants/guardians.

#### 2.1.2 MRI Protocol

The GE Signa HDxt 3.0 T superconducting magnetic resonance imaging system was used for magnetic resonance diffusion‐weighted imaging (DWI) and fluid‐attenuated inversion recovery (FLAIR).

#### 2.1.3 Serum Analysis

Fasting venous blood (5 mL) was collected, clotted at room temperature for 20 min, and centrifuged to isolate serum. Serum iron was quantified using a commercial kit (Pointe Scientific, USA), whereas GSH‐Px and GSH levels were assessed with kits from Nanjing Jiancheng Bioengineering Institute.

### 2.2 Experimental Animals

Male C57BL/6 mice (20 ± 5 g) were obtained from the Laboratory Animal Center of Nantong University (SYXK 2022‐0046). Animals were housed in a specific pathogen‐free (SPF) facility maintained at 23°C–25°C with a 12 h light/dark cycle and free access to food/water. All experimental protocols were approved by the Institutional Animal Care and Use Committee of Nantong University (S20221113‐903).

### 2.3 CPZ Feeding Induced Brain Demyelination Model and Groups

This research group conducted basic experiments according to the improved CPZ demyelinating model method [32]. Male C57/BL6 mice (5‐week‐old) were fed 0.2% cuprizone (CPZ, Merck C9012) for 5 weeks to induce demyelination. The HDAC3 inhibitor RGFP966 was used for myelin protection at doses similar to those used in recent demyelinating models [29, 33]. Dissolve HDAC3 inhibitor RGFP966 (Selleckchem, S7229) (10 mg/kg) [30] in 5% DMSO (MCE, HY‐Y0302) + 40% PEG300 (Selleckchem, S6704) + 10% Tween80 (Selleck, S67020) + 45% normal saline and administer by intraperitoneal injection on Days 1 to 5. The specific group of mice and the experimental time point can be seen in Figure S1. To induce HDAC3 overexpression, 1 μL of AAV vector encoding HDAC3 (1.0 × 10^12^ viral genomes/mL) was stereotaxically injected into the corpus callosum at a rate of 0.2 μL/min (coordinates relative to bregma: anterior–posterior +1.0 mm, medial–lateral +0.3 mm, dorsal–ventral −2.2 mm). Brain tissues were collected 3 weeks post‐injection, corresponding to the peak period of viral transgene expression.

### 2.4 Behavioral Assessments

#### 2.4.1 Rotarod Test

The experiment was conducted on Days 33 to 35 following CPZ induction. The detection process follows the steps in our previous research [34]. In the rotarod test, the trial was terminated when a mouse either fell or remained suspended without forward movement for two consecutive rotations. The time each mouse remained on the rotating rod was precisely recorded and used to assess motor coordination and balance.

#### 2.4.2 Morris Water Maze (MWM)

The experiment was conducted between Days 30 and 35 following CPZ induction. The water maze detection process follows the steps in our previous research [34]. During the acquisition phase (Days 1–4), the latency to locate the hidden platform (maximum 90 s), swimming path, and speed were recorded for each mouse. On Day 5 (probe trial), the platform was removed, and the swimming trajectory and time spent in the target quadrant within a 60‐s period were measured to evaluate spatial memory retention.

#### 2.4.3 Novel Object Recognition (NOR)

This experiment was conducted on Day 35 of the CPZ‐induced model. The NOR detection process follows the steps in Zheng's research [35]. Discrimination scores ranged from −1 to +1, with negative values indicating a preference for the familiar object and positive values indicating a preference for the novel object.

### 2.5 Sample Collection

The mice were anesthetized by 2.5% Avertin intraperitoneally and injected with 0.9% normal saline. When the liver turned white, the heart was injected with 4% PFA, and the perfusion was stopped when the tail of the mice was bent and cocked. The mouse brain tissue was infiltrated at 4°C overnight after PFA and dehydrated with 20% sucrose for 1 day and 30% sucrose for another day. The brain tissue was sliced in a frozen microtome (Leica, GER) at a constant temperature of −20°C with a thickness of 15 μm.

#### 2.5.1 Luxol Fast Blue Staining (LFB)

The frozen sections were dyed with 60°C fast blue solution (American MasterTech, USA, KTLFB) for 2 h, rinsed with ddH_2_O, followed by ethanol decolorization, 0.05% lithium carbonate (RHAWN, 554‐13‐2) color separation and gradient ethanol dehydration, and then made transparent with xylene and sealed with neutral gum. It is dried and imaged under a microscope.

#### 2.5.2 Diaminobenzidine (DAB) Enhanced Iron Staining

Frozen sections (15 μm) were thermally stabilized at 37°C for 1 h prior to fixation with 70% ethanol. After three 2 min rinses in ddH_2_O, sections underwent agitated incubation in 1% potassium ferricyanide solution to facilitate Fe^3+^‐dependent Prussian blue precipitation. Endogenous peroxidase activity was blocked by light‐protected incubation with 0.3% H_2_O_2_ in methanol, followed by chromogenic development in DAB solution (Beyotime, P0202) under microscopic monitoring (≤ 10 min) until optimal brown precipitate formation. Nuclear counterstaining was performed using hematoxylin with subsequent alkaline differentiation for cytoplasmic clarity. Dehydration through an ascending ethanol series preceded triple xylene clearing. Sections were permanently mounted with neutral gum for histological analysis.

#### 2.5.3 Immunofluorescence (IF)

Frozen sections underwent PBS washing followed by permeabilization with 1% Triton X‐100 for 1 h. After blocking with 5% donkey serum (1 h), sections were incubated with primary antibodies at 4°C overnight. Species‐matched fluorescent secondary antibodies were applied for 1 h at room temperature. The nuclei were labeled with DAPI, the slides were bleached to the slides and then sealed and dried. Confocal imaging was performed in 48 h. Cellular immunofluorescence was performed directly in the cell culture plate, and cells were immobilized with pre‐cooled 4% PFA after the cell medium was cleaned. The other steps are the same as tissue. Antibody information used in the experiment: MBP (Abcam, UK, ab40390, 1:300), SMI32 (Biolegend, 801701, 1:200), APC (Abcam, ab16794, 1:200), HDAC3 (Abcam, Ab137704, 1:200), NG2 (Santa Cruz, sc‐33666, 1:200), Olig2 (Millipore, AB9610, 1:150), BrdU (Abcam, ab6326, 1:200), FTH (Novus, NBP1‐31944, 1:200), TFRC (Thermo, USA, 136800, 1:200), MAP2 (Cell signaling, 8707S, 1:150), NeuN (Cell signaling, D3S3I, 1:200), DAPI (Beyotime, C1006, 1:2500). AlexaFluor488 DAR (Jackson, 711‐545‐152, 1:1000), Cy3 DAM (Jackson, 715‐165‐151, 1:1000), Cy3 DAR (Jackson, 711‐165‐152, 1:1000).

### 2.6 Western Blotting

Brain white matter lysates were centrifuged to collect supernatants. Protein concentrations were determined (Thermo Fisher, 23225), and adjusted to 2 μg/μL. A gel with protein molecular weight was prepared and electrophoresis was carried out at a constant voltage of 120 V (Tanon, CN). Samples underwent SDS‐PAGE and wet‐transferred to PVDF membranes (Millipore IPVH00010) at 300 mA for 60 min, 4°C. Membranes were blocked with 5% skim milk for 2 h, incubated with primary antibodies (Table S1) at 4°C overnight, then HRP‐conjugated secondary antibodies for 2 h. Protein bands were visualized via chemiluminescence and quantified using ImageJ.

### 2.7 Detection of Lipid Peroxidation Indexes in Mice

After anesthesia, the mice were injected into the heart with normal saline, and the brain white matter tissue samples were taken, which were cut and ground on ice. After resting, the supernatant was taken by centrifugation for detection of malondialdehyde (MDA) (Solarbio, bc0025), superoxide dismutase (SOD) (Solarbio, bc5165) and glutathione (GSH) (Solarbio, bc1175) according to the instructions.

### 2.8 Enzyme‐Linked Immunosorbent Assay (ELISA) for Mouse Transferrin

Mouse transferrin (TF) levels were measured using a commercial enzyme‐linked immunosorbent assay (ELISA) kit (Elabscience, E‐EL‐M1184, Elabscience Biotechnology Co. Ltd., Wuhan, Hubei, China).

### 2.9 CUT&Tag‐ Quantitative Real‐Time PCR

The nucleus of white matter tissue of mouse brain was extracted (Solarbio, SN0020). Hyperactive Universal CUT&Tag Assay Kit for iiumina Pro (Vazyme, TD904) will be used to prepare samples, part of which will be used for quantitative polymerase chain reaction detection (qPCR), and the other part will be amplified and sent to Novogene for sequencing. The sequencing data will be decoded on the cloud platform of Vazyme for bioinformatics analysis. The decoded file is visualized using IGV software.

### 2.10 Quantitative Real‐Time PCR (qPCR)

Tissues were homogenized in 200 μL TRIzol. Phase separation was achieved using chloroform followed by centrifugation. RNA was precipitated with isopropyl alcohol, washed three times with 75% ethanol, air‐dried, and resuspended in 15 μL DEPC‐treated water after 60°C denaturation (10 min). Reverse transcription was performed using a commercial kit (Vazyme, Q141‐02). qPCR was conducted on a ROCGENE Archimed X6 system with primers listed in Table S2.

### 2.11 In Vitro Demyelination Models

#### 2.11.1 Neuronal Model

Cerebral cortices from SD rats were dissected, with meninges and hippocampus removed. Tissues were digested with trypsin, dissociated into single‐cell suspensions, and plated in 12‐well plates. Three experimental groups were established. Control: Neurons maintained in a 5% CO_2_ incubator at 37°C without intervention. Iron overload model: Neurons treated with 100 μM FeCl_3_ for 24 h. HDAC3 inhibition: Neurons co‐treated with 100 μM FeCl_3_ and 10 μM RGFP966 for 24 h. Cells were harvested post‐treatment for subsequent analyses.

#### 2.11.2 Oligodendrocyte Precursor Cell (OPC) Model

Single‐cell suspension is prepared in the same way as neurons. Cells were resuspended in culture medium and seeded into lysine‐coated T75 vials. After 8 days of culture (37°C, 5% CO_2_), OPCs were purified via microglial depletion. Three experimental groups were established. Control: Untreated OPCs. Iron overload: OPCs treated with 100 μM FeCl_3_ for 24 h. HDAC3 inhibition: OPCs co‐treated with 100 μM FeCl_3_ and 10 μM RGFP966 for 24 h. Cells were harvested 24–48 h post‐treatment for other analyses.

### 2.12 Statistical Analysis

Statistical data were analyzed by GraphPad Prism 8.0 software, and the values were expressed as Mean ± SEM. Prior to statistical testing, data distribution was assessed using the Shapiro–Wilk test for normality and Levene's test for homogeneity of variance. If both normality and homogeneity of variance were satisfied, parametric tests were applied. Comparisons between two groups were conducted using either the independent samples *t*‐test or the paired *t*‐test, as appropriate. Comparisons among three or more groups were analyzed using one‐way ANOVA followed by Tukey's post hoc test. For data that did not meet the assumptions of normality or equal variance, appropriate non‐parametric tests were used, including the Mann–Whitney *U* test for two‐group comparisons or the Kruskal–Wallis test with Dunn's post hoc test for multiple groups. *p* < 0.05 was considered statistically significant.

## 3 Results

### 3.1 Clinical WMI Patients With White Matter Degeneration Accompanied by Iron Deposition

**FIGURE 1:**
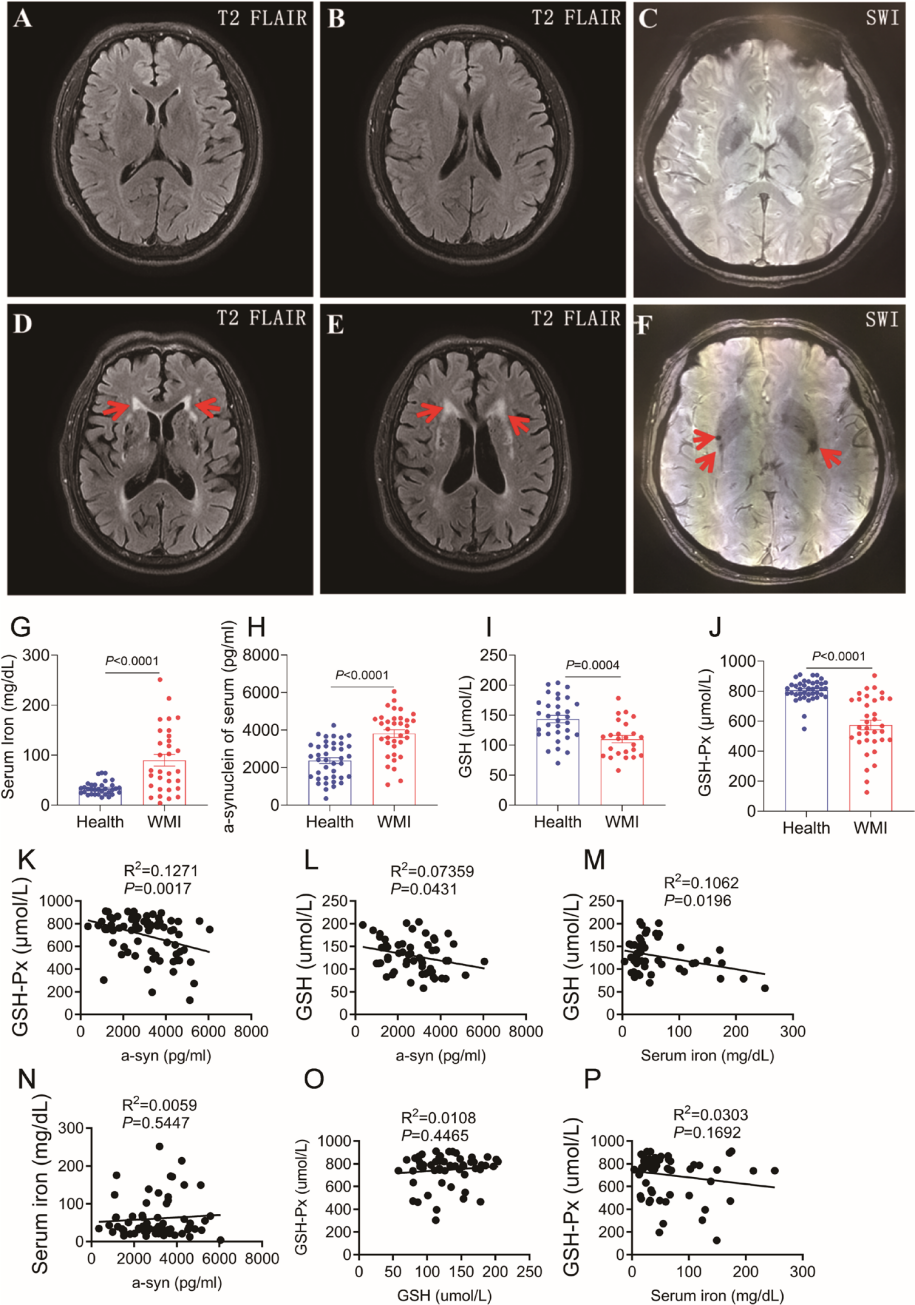
Magnetic resonance imaging and serum detection of white matter injury (WMI) patients. (A, B) T2‐weighted imaging of horizontal head in healthy volunteers. (C) Head Susceptibility weighted imaging (SWI) in healthy volunteers. (D, E) T2‐weighted imaging of head in patients with WMI. The red arrow is a speckled high signal in the white matter area. (F) Head SWI imaging of WMI patients, with red arrows indicating point‐like extremely low signals in bilateral basal ganglia. (G–J) Determination of serum iron content, α‐synuclein (α‐syn) concentration, Glutathione (GSH) content and glutathione peroxidase (GSH‐Px) concentration in healthy volunteers and WMI patients. *n* = 31. (K) Correlation detection of serum components of WMI patients, GSH‐Px/α‐syn; GSH/α‐syn; GSH/serum iron; serum iron/α‐syn; GSH‐Px/α‐syn; GSH‐Px/serum iron.

To investigate the relationship between WMI and disorders of iron metabolism, we recruited healthy volunteers and patients with WMI in our clinic in a case–control study, tested the extent of white matter damage and the presence of iron deposition by imaging, and serum iron levels and antioxidant system levels by serology. The primary inclusion criteria were patients with white matter injury aged 35–65 years. As shown in Figure 1A–C, no obvious abnormal signals were found in the brain parenchyma of healthy volunteers. In T2‐weighted imaging, multiple punctate high signals appeared in the bilateral radial crowns, centers of the semiovals, and subcortical white matter regions in patients with WMI, suggesting white matter degeneration (Figure 1D,E). SWI sequence shows a very low signal in the basal ganglia of both sides, indicating iron deposition (Figure 1F). Serum iron content in WMI patients was significantly higher than that in healthy controls (Figure 1G). α‐synuclein (α‐syn) is a key protein in the pathogenesis of Parkinson's disease. Serum α‐syn was significantly increased in WMI patients (Figure 1H), whereas the contents of glutathione peroxidase (GSH‐Px) and glutathione (GSH) decreased in both patients compared with the control group (Figure 1I,J), suggesting that the antioxidant system of the patients was weakened. In addition, α‐syn concentration was positively correlated with GSH and GSH‐Px concentration, and serum iron concentration was also significantly positively correlated with GSH concentration (Figure 1K–M), but serum iron was almost not correlated with α‐syn and GSH‐Px. There was no statistically significant linear correlation between GSH concentration and GSH‐Px concentration (Figure 1N–P). These results suggest that the glutathione metabolic system is dysregulated and the antioxidant system is weakened after WMI.

### 3.2 CPZ‐Induced White Matter Demyelination in Mice Leads to Neurological Dysfunction and Dysregulation of Cerebral Iron Metabolism

**FIGURE 2:**
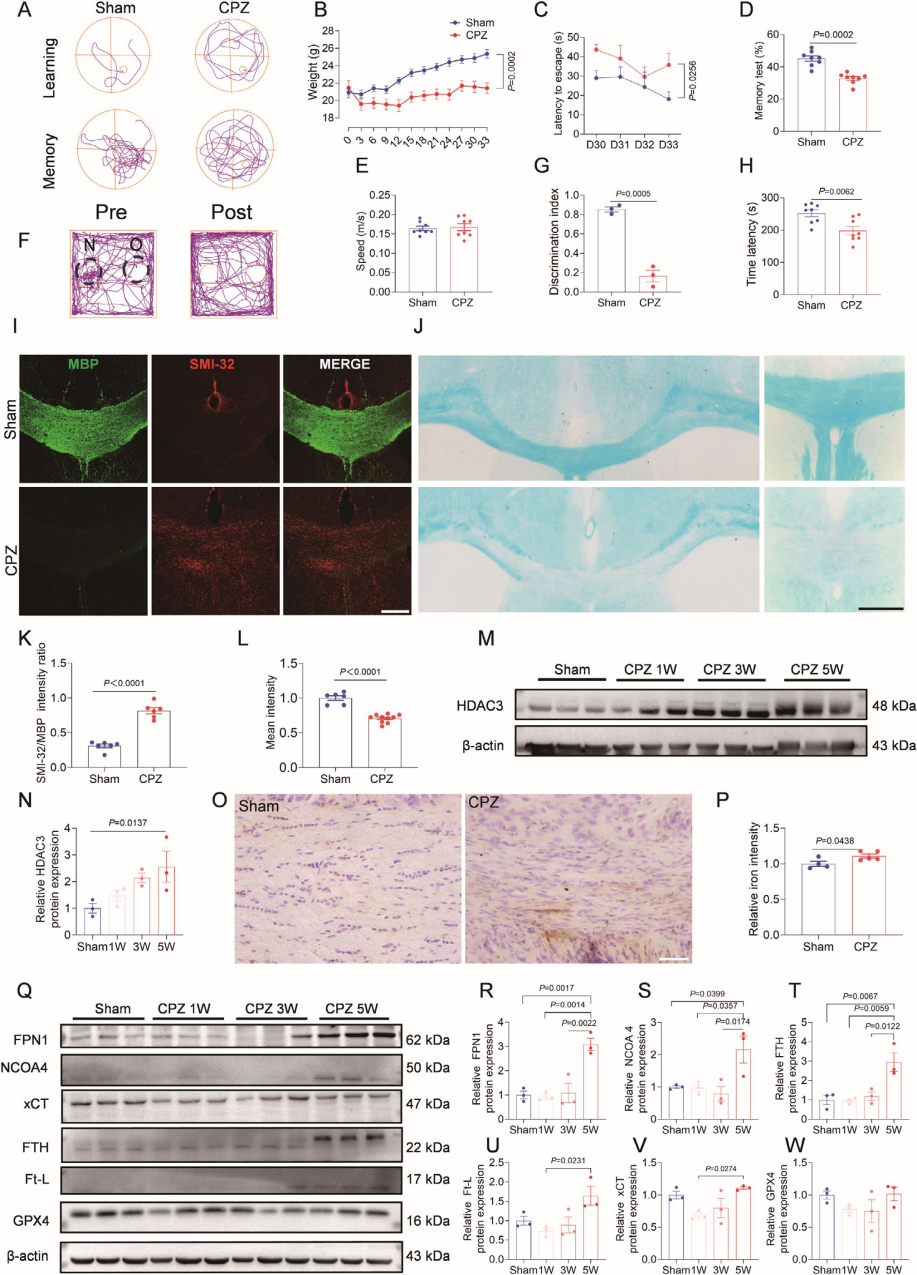
Cuprizone (CPZ)‐induced demyelination impairs nerve function in mouse. (A) Morris Water Maze (MWM) swimming trail diagram. (B) Weight changes of mouse during modeling. *n* = 11–12. (C) MWM escape latency. The time to find the target platform from Day 30 to Day 33 of demyelination. *n* = 5. (D) MWM memory ability. The proportion of time spent in the quadrant where the target platform was located on the 34 day of demyelination; (E) Swimming speed during MWM. *n* = 8. (F) Novel object recognition (NOR) mouse representative trajectory map, pre is before modeling, post is after modeling, N means new object, O means old object. (G) NOR discrimination index. *n* = 3. (H) The length of time the mouse remained on the rotarod was measured during the final week of demyelination. *n* = 8. (I) Representative double immunofluorescence images of MBP‐positive myelin and SMI32‐positive non‐phospholipid filament H protein in the corpus callosum (CC) region. Scale bar = 250 μm. (J) LFB staining in CC region. Scale bar = 200 μm. (K) Quantitative statistical chart of SMI32/MBP fluorescence intensity ratio; *n* = 6. (L) Quantitative statistical chart of average strength of LFB; *n* = 6–10. (M, N) Representative western blot bands and quantification of Histone deacetylase 3 (HDAC3) protein expression levels after demyelination, respectively in sham operation group, CPZ feeding for 1 week, 3 weeks and 5 weeks. *n* = 3. (O) Representative Diaminobenzidine (DAB) enhanced iron staining in CC of 5‐week demyelinating mouse. The white solid line in the lower right corner represents. Scale bar = 200 μm. (P) Quantitative statistical diagram of DAB enhanced iron staining intensity of 5‐week demyelinating mouse. *n* = 4–5. (Q) Representative western blot bands of ferroportin 1 (FPN1), nuclear receptor coactivator 4 (NCOA4), ferritin heavy chain (FTH), ferritin light chain (Ft‐L), glutathione peroxidase 4 (GPX4) and solute carrier family 7 member 11 (xCT) in the white matter of mouse brain after demyelination in 1 week, 3 weeks and 5 weeks. (R‐W) Quantitative analysis of protein expression levels of FPN1, NCOA4, FTH, Ft‐L, xCT, GPX4 in CC region. *n* = 4–5.

To simulate clinical WMI patients, studies were performed according to a modified CPZ demyelination modeling approach. The CPZ model reproduced progressive iron accumulation and time‐dependent demyelination, reflecting clinical WMI pathology. The effects of the CPZ‐induced demyelination model on neurological function in mice were assessed by a behavioral testing system (Figure 2A). CPZ‐fed mice exhibited growth retardation (Figure 2B), and multifunctional neurological deficits: prolonged escape latency in MWM indicated cognitive impairment. The effect of motor function interference on cognitive assessment was excluded (Figure 2C–E), reduced novel object exploration confirmed short‐term memory deficits (Figure 2F,G), and decreased rotarod performance revealed motor coordination dysfunction (Figure 2H). Histological analysis at Week 5 showed severe myelin disruption, evidenced by reduced MBP expression, elevated SMI32 levels (Figure 2I,K), and disrupted myelin integrity in LFB staining (Figure 2J,L). HDAC3 expression progressively increased during CPZ exposure, peaking at Week 5 (Figure 2M,N). Concurrently, DAB‐enhanced staining revealed iron deposition in the corpus callosum (Figure 2O,P), whereas upregulated iron metabolism proteins (FPN1, NCOA4, FtL, FTH) confirmed white matter iron overload (Figure 2Q–U). Ferroptosis markers GPX4 and xCT showed progressive suppression post‐demyelination (Figure 2V,W). These findings demonstrate that CPZ‐induced demyelination recapitulates clinical WMI pathology, manifesting as impaired learning/memory and disrupted white matter iron homeostasis consistent with patient observations.

### 3.3 HDAC3 Inhibition Restores Myelin Integrity by Enhancing OLGs Maturation and Suppressing OPCs Proliferation

**FIGURE 3:**
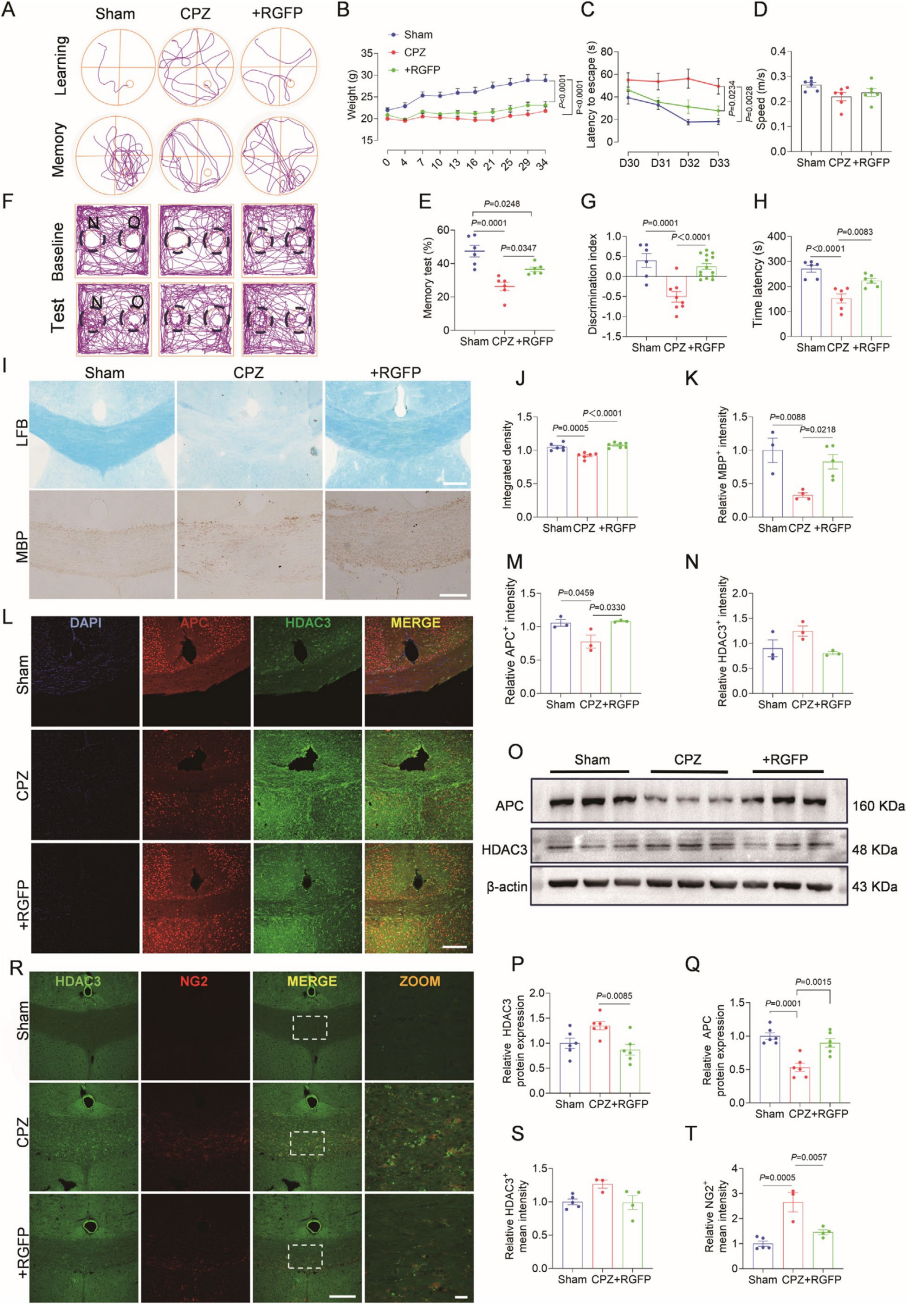
HDAC3 inhibition protects neural function and maintains myelin integrity in mouse. (A) MWM swimming tracks of mouse. (B) Changes of body weight during demyelination of mouse. *n* = 5–8. (C) MWM escape latency. The time for mouse in each group to find the target platform on Days 30 to 34 of demyelination. *n* = 8. (D) MWM memory ability, the proportion of time spent in the quadrant where the target platform is located on Day 33. (E) MWM swimming speed; *n* = 6. (F) NOR trajectory diagram, the above is the representative trajectory diagram before modeling, the following is the representative trajectory diagram after modeling. (G) NOR discrimination index. *n* = 6–14. (H) Rotarod test for the fifth week of demyelination, and the ordinate represents the time the mouse remain on the rotarod. *n* = 3–8. (I) LFB and DAB enhanced immunohistochemistry (IHC) staining in CC region. Scale bar = 200 μm. (J) Quantitative statistical chart of LFB staining intensity. *n* = 6. (K) Quantitative statistical diagram of MBP IHC staining intensity. *n* = 3–5. (L) Representative immunofluorescence images of APC/HDAC3 co‐labeling in CC region. DAPI (blue to mark nucleus), APC (red to mark mature oligodendrocytes(OLGs) and HDAC3 (green to mark HDAC3). Scale bar = 250 μm. (M, N) Quantitative analysis of co‐localized fluorescence intensity was performed using ImageJ where regions of CC containing both APC‐positive OLGs and HDAC3‐positive cells. *n* = 3. (O) Representative western blot bands of APC and HDAC3 in sham group, CPZ group and RGFP966 group. (P–Q) Quantitative analysis of protein expression levels of HDAC3 and APC in the CC region. *n* = 3–6. (R) Representative immunofluorescence images of HDAC3/NG2 co‐labeling in CC region. HDAC3 (green, marking HDAC3) and NG2 (red, marking oligodendrocyte precursor cells) are locally enlarged by ZOOM. Scale bar = 250 μm, 50 μm. (S, T) Quantification of co‐localized fluorescence intensity of HDAC3‐positive cells and NG2‐positive OPCs in the CC region. *n* = 3–5.

HDAC3 was observed to be elevated in the white matter of mouse brain after demyelination. To explore the mechanism of the effect of HDAC3 on OLGs during demyelination, an intervention using the HDAC3 inhibitor RGFP966 was performed. The neuroprotective effect of RGFP966 on mice was first examined (Figure 3A). RGFP966 treated mice exhibited improved weight gain (Figure 3B), reduced MWM escape latency, and increased target quadrant preference without altered swim speed (Figure 3C–E). Enhanced novel object exploration and rotarod performance confirmed cognitive and motor coordination recovery (Figure 3F–H). Histologically, RGFP966 preserved myelin integrity, evidenced by restored LFB staining intensity and MBP expression (Figure 3I–K). Co‐localization analysis revealed HDAC3 overexpression inversely correlated with mature oligodendrocyte depletion in CPZ mice—a trend reversed by HDAC3 inhibition (Figure 3L–N), corroborated by Western blot quantification (Figure 3O–Q). Dual immunofluorescence demonstrated HDAC3 co‐expression with proliferating OPCs in CPZ lesions, which RGFP966 suppressed (Figure 3R–T). These results suggest that HDAC3 aggravates WMI by inhibiting OLGs maturation and promoting abnormal proliferation of OPCs in demyelinating pathology, whereas inhibiting HDAC3 regulates oligodendrocyte homeostasis to improve nerve function and maintain myelin integrity.

### 3.4 HDAC3 Inhibition Promotes OPC‐To‐OLG Maturation and Remyelination

**FIGURE 4:**
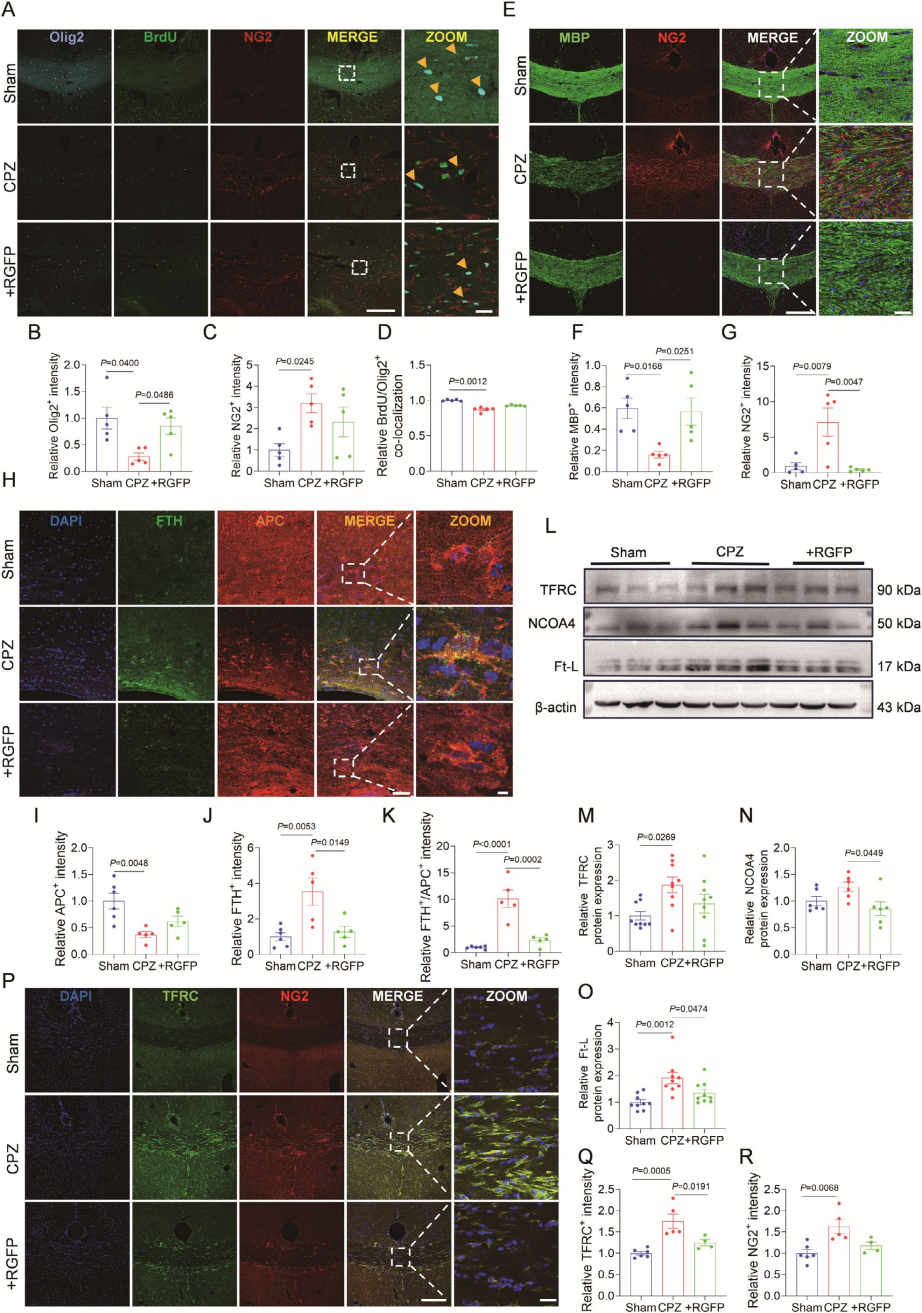
Inhibition of HDAC3 accelerates the differentiation of OPCs into OLGs and promotes remyelination. (A) Representative immunofluorescence images of OLIG2/BrdU/NG2 co‐labeling in CC region. Olig2 (blue for OLGs), BrdU (green for 5‐bromo‐2′‐deoxyuridine) and NG2 (red for OPCs). Scale bar = 250 μm, 25 μm. (B‐D) Quantification of co‐localized fluorescence intensity of Olig2‐positive cells, NG2‐positive OPCs, BrdU/Olig2‐positive cells ratio in each group. *n* = 5. (E) Representative immunofluorescence images of MBP/NG2 in the CC region. Scale bar = 250 μm, 50 μm. (F, G) Quantification of co‐localized fluorescence intensity of MBP‐positive myelin and NG2‐positive OPCs. *n* = 5. (H) Representative immunofluorescence images of FTH/APC in the CC region. FTH (green to mark heavy chain ferritin) and APC (red to mark mature oligodendrocytes). Scale bar = 25 μm, 5 μm. (I–K) Quantitative analysis of co‐localized fluorescence intensity was performed using ImageJ where regions of CC containing both APC‐positive OLGs and FTH‐positive cells. *n* = 5–6. (L) Representative western blot bands of TFRC, NCOA4 and Ft‐L in RGFP966 after demyelination. They were divided into sham operation group, demyelination group and RGFP966 myelin protection group. (M–O) Quantitative analysis of protein expression levels of TFRC, NCOA4 and Ft‐L bands in CC region. *n* = 3–6. (P) Representative immunofluorescence images of NG2/TFRC in CC region. Scale bar = 250 μm, 50 μm. (Q,R) Quantitative statistics of fluorescence intensity of TFRC‐positive cells and NG2‐positive OPCs in each group. *n* = 4–6.

The effects of HDAC3 on the maturation of OLGs and the proliferation of OPCs have been observed. To clarify the regenerative mechanism of the inhibition of HDAC3 on oligodendrocyte lineage and myelin protection, co‐localization analysis of Olig2/BrdU/NG2 was performed, and the dynamic regulatory role of HDAC3 in oligodendrocytes was explored by intraperitoneal injection of BrdU during 5 weeks of demyelination in mice. CPZ‐treated mice exhibited a decrease in mature oligodendrocytes with concomitant proliferation of OPCs, whereas inhibition of HDAC3 by RGFP966 increased Olig2/BrdU co‐labeling of cells with concomitant inhibition of over‐proliferation of OPCs (Figure 4A–D). The effect of HDAC3 on the proliferation and differentiation of oligodendrocyte lineage was further investigated by MBP and NG2 (Figure 4E). It was found that MBP staining decreased and the NG2 proliferation level increased in the CPZ group, indicating that the level of proliferation, differentiation, and maturation of OPCs into complete myelin decreased. However, inhibition of HDAC3 by RGFP966 increased the level of differentiation and maturation, thus promoting myelin regeneration in the white matter corpus callosum region of mice (Figure 4F,G). It was demonstrated that inhibition of HDAC3 enhanced the differentiation of OPCs to OLGs and promoted myelin regeneration.

### 3.5 HDAC3 Inhibition Alleviates Iron Overload and Oligodendrocyte Lineage Dysfunction During Demyelination

Further immunohistochemical analysis revealed that FTH was highly expressed in mature oligodendrocytes of CPZ‐treated mice, correlating with iron overload and oligodendrocyte loss. Notably, HDAC3 inhibition alleviated both iron accumulation and oligodendrocyte degeneration (Figure 4H–K). Western blotting confirmed that inhibition of HDAC3 downregulated the abnormally high expression of NCOA4, Ft‐L, and TFRC, which are key regulators affecting iron storage and transferrin uptake and are associated with ferroptosis [36, 37]. Co‐localization of TFRC/NG2 in demyelination suggests that iron drives OPC proliferation, whereas inhibition of HDAC3 slows this proliferation while promoting differentiation (Figure 4P–R). This result further revealed that the abnormal proliferation of OPCs is related to the disorder of iron metabolism, and inhibiting HDAC3 improved the differentiation disorder of OPCs caused by iron deposition during demyelination.

### 3.6 HDAC3 Inhibition Alleviates Demyelination‐Associated Ferroptosis by Restoring Iron Homeostasis and Suppressing Lipid Peroxidation

**FIGURE 5:**
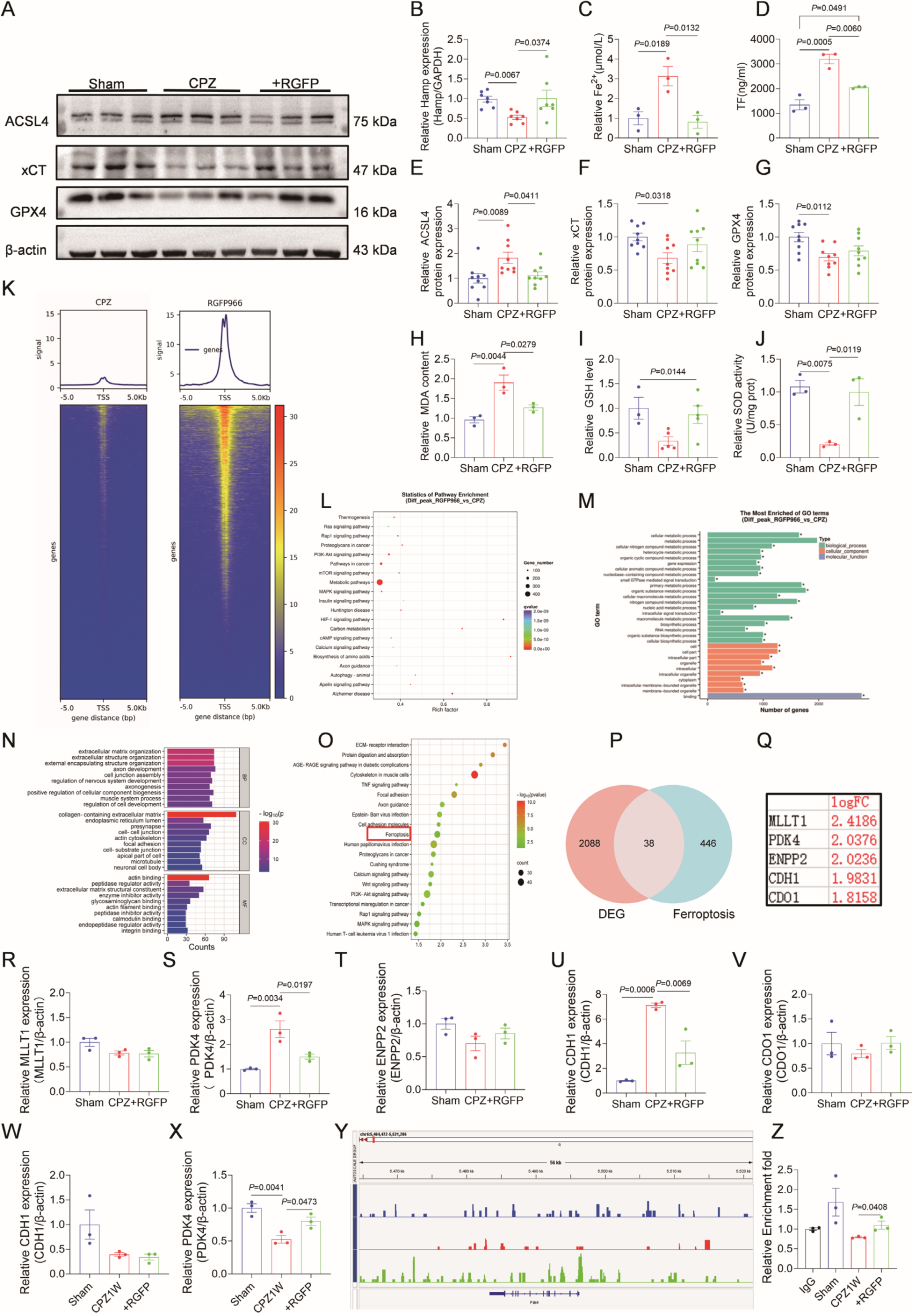
Inhibition of HDAC3 regulates iron metabolism in HDAC3‐PDK4 axis and improves ferroptosis by lipid peroxidation. (A) Representative western blot bands of ACSL4, xCT and GPX4 in sham, CPZ and RGFP966 intervention group. (B) Quantification of hepcidin antimicrobial peptide (*Hamp*) mRNA expression examined by quantitative real‐time PCR (qPCR) in white matter after RGFP966 intervention. (C) Fe^2+^ expression in white matter after RGFP966 intervention. (D) Quantification of the transferrin (TF) levels by Enzyme‐Linked Immunosorbent Assay (ELISA) at 5 weeks CPZ‐modeling in CC region. *n* = 3–7. (E–G) Quantitative analysis of protein expression levels of ACSL4, xCT and GPX4 in CC region. *n* = 3–6. (H) Malondialdehyde (MDA) was detected in the CC region of mice. (I) Quantitative analysis of GSH expression levels in white matter of mouse in each group; (J) The activity level of superoxide dismutase (SOD) in the white matter of mouse in each group. *n* = 3–5. (K) CUT&Tag sequencing Reads enrichment map. The graph above shows the gene region in horizontal coordinates and the signal enrichment intensity in vertical coordinates. The following is a heat map. The horizontal coordinate represents 5 kb upstream and 5 kb downstream of the transcription initiation site (TSS), the vertical coordinate represents genes, and the color represents the coverage of reads at corresponding locations. The redder the color, the higher the coverage of reads at that location. (L) In the KEGG enrichment results of peak‐related genes, select the pathway with the most significant enrichment and show it in the figure. The enrichment of KEGG Pathway was measured by RichFactor, Qvalue and the number of genes enriched in this pathway. (M) Peak GO enrichment analysis of CPZ group and RGFP966 group. (N, O) KEGG analysis diagram and GO enrichment analysis of NCBI dataset GSE151306. (P, Q) The top five genes with high logFC values among 38 intersection genes in NCBI dataset and ferroptosis gene dataset. (R–V) Quantification of *PDK4, ENPP2, MLLT1, CDH1* and *CDO1* mRNA expression examined by qPCR in the white matter of mouse after 5 weeks of demyelination. *n* = 3. (W–X) Quantification of *CDH1* and *PDK4* mRNA expression examined by qPCR in the CC region of mouse after 1 week of intervention with RGFP966. (Y) Reads expression of *PDK4* in IGV visual CUT&Tag sequencing data. (Z) Quantification of the binding effect of HDAC3 and PDK4 expression examined by CUT&Tag‐qPCR after demyelination for 1 week. *n* = 3.

The oxidative state of iron, particularly Fe^2+^, critically drives ferroptosis via Fenton reaction‐mediated oxidative stress. In order to determine the oxidation status of iron in the white matter of mice after demyelination, lipid peroxidation related indexes were detected (Figure 5A). Demyelination in CPZ mice induced iron redox imbalance, characterized by decreased Hamp mRNA and elevated transferrin (TF) and Fe^2+^ levels in the white matter—effects reversed by HDAC3 inhibition with RGFP966 (Figure 5B–D). Western blot results showed an increase in ACSL4 after demyelination, suggesting increased activation of intracellular long‐chain fatty acids, thereby promoting lipid peroxidation, whereas RGFP966 reduced the level (Figure 5E). Protein levels of GPX4 and xCT were reduced after CPZ, reflecting reduced cellular antioxidant capacity and increased cellular sensitivity to ferroptosis, and were on the rise after the inhibition of HDAC3 (Figure 5F,G). Lipid ROS plays a key role in ferroptosis, and it has been reported that increased SOD inhibits ROS levels in vivo [38]. HDAC3 inhibition attenuated lipid peroxide accumulation, restoring redox balance through reduced MDA and rescued GSH/SOD activity (Figure 5H–J). These findings suggest that HDAC3 is upregulated after demyelination, disrupting iron ion state homeostasis and amplifying lipid peroxidation, thereby triggering ferroptosis. Inhibition of HDAC3 by RGFP966 restores iron metabolism and enhances the antioxidant defense of oligodendrocytes, thereby attenuating ferroptosis.

### 3.7 HDAC3 Drives Metabolic Reprogramming in Demyelination via Epigenetic Regulation of PDK4‐Mediated Ferroptosis

To identify HDAC3‐regulated targets in demyelination, we performed CUT&Tag sequencing on white matter from CPZ‐treated and RGFP966‐protected mice (Figure 5K). KEGG/GO analyses revealed HDAC3‐associated genes were enriched in metabolic pathways (Figure 5L,M). Differential genes were screened by bioinformatics analysis of the demyelination sample dataset from the NCBI database. KEGG analysis revealed the presence of a ferroptosis pathway (Figure 5N,O). After intersection of differential genes and the ferroptosis gene dataset, 38 genes were obtained, and 5 genes with large differences in logFC values were selected for verification to identify key target genes (Figure 5P,Q). Both *CDH1* and *PDK4* were upregulated at 5 weeks of demyelination (Figure 5R–V). Since the expression levels of HDAC3 and ferroptosis‐associated proteins increased in a time‐dependent manner, the increase started at 1 week of demyelination. Therefore, samples from 1 week of demyelination were examined. At 1 week of demyelination, the expression level of *PDK4* was low, and RGFP966 negatively regulated its expression level, but not the *CDH1* gene (Figure 5W,X). IGV analysis of CUT&Tag sequencing data revealed diminished HDAC3‐PDK4 genomic binding at 1 week post‐demyelination, with PDK4‐associated DNA fragments showing low enrichment, whereas RGFP966 treatment elevated PDK4 chromatin accessibility and peak intensity (Figure 5Y). qPCR validation of CUT&Tag products confirmed HDAC3‐PDK4 interaction. At Week 1 post‐demyelination, both PDK4 expression and HDAC3‐PDK4 binding were reduced, whereas HDAC3 inhibition reversed PDK4 suppression (Figure 5Z). These data establish that HDAC3 drives ferroptosis via epigenetic activation of PDK4, inducing metabolic reprogramming and iron‐mediated oligodendrocyte death. HDAC3 inhibition restores PDK4 homeostasis, mitigating oxidative damage and enabling myelin repair.

### 3.8 HDAC3 Drives Ferroptosis as a Core Pathogenic Driver

**FIGURE 6:**
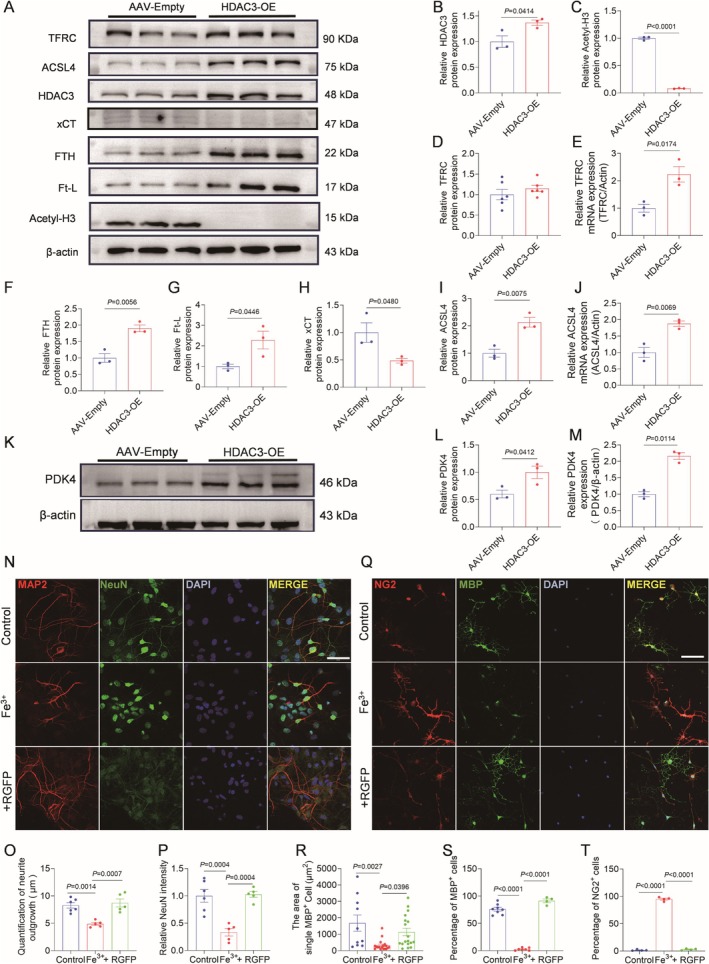
Inhibition of HDAC3 negatively regulates ferroptosis. (A) Representative western blot bands of TFRC, ACSL4, HDAC3, xCT, FTH, Ft‐L, Acetyl‐H3 in CC region after stereotaxic injection of HDAC3 overexpressed adeno‐associated virus (AAV). AAV‐Empty was used as an empty viral vector control in adeno‐associated virus infection experiments. (B, D, C, F, G, H, I) Quantitative analysis of protein expression levels of TFRC, ACSL4, HDAC3, xCT, FTH, Ft‐L, Acetyl‐H3 in CC region. *n* = 3. (E, J) Quantification of *TFRC* and *ACSL4* mRNA expression examined by qPCR in CC region after stereotaxic injection of HDAC3‐AAV. *n* = 3. (H) Representative western blot bands of PDK4 protein in white matter of mouse after HDAC3‐AAV injection; (L) Quantitative analysis of protein expression levels of PDK4 in the CC region. *n* = 3. (M) Quantification of *PDK4* mRNA expression examined by qPCR in CC region after stereotaxic injection of HDAC3‐AAV. *n* = 3. (N) Representative cellular immunofluorescence images of MAP2/NeuN co‐labeling in a primary neuronal iron overload model. MAP2 (red marking neuronal dendrites), NeuN (green marking neuron body). Scale bar = 50 μm. (O, P) Quantitative statistics of neuronal process length and neuronal cell body fluorescence intensity of each labeled group. *n* = 6. (Q) Representative cellular immunofluorescence images of MBP/NG2 co‐labeling in a OPCs iron overload model. Scale bar = 50 μm. (R) Quantitative analysis of single area of MBP‐positive cells in each group. (S) Quantitative analysis of the percentage of MBP labeled OLGs in all cells in each group. (T) Quantitative analysis of the percentage of NG2‐positive OPCs in each group. *n* = 6.

To confirm the direct role of HDAC3 in ferroptosis, overexpression of HDAC3 adeno‐associated virus was injected into the corpus callosum region of the mouse brain using stereotactic injection, and samples were taken at Week 3. Western blotting analysis showed that the expression level of ferroptosis‐associated proteins was markedly elevated after HDAC3 overexpression (Figure 6A). The increase of HDAC3 and the decrease of acetylated H3 levels confirmed the effectiveness of HDAC3 overexpressed AAV construction (Figure 6B,C). Although the TFRC protein remained unchanged (Figure 6D), its mRNA levels increased (Figure 6E) and the expression of FTH and Ft‐L proteins increased (Figure 6F,G). The results indicated that overload of HDAC3 affected iron metabolism. HDAC3 overexpression inhibited xCT protein expression (Figure 6H), and both protein levels and mRNA expression of ACSL4 increased. These results suggest that HDAC3 overload affects lipid peroxidation levels and reduces antioxidant levels. The expression levels of PDK4 protein and mRNA were increased after HDAC3 overexpression (Figure 6K–M). This confirms the previously discovered HDAC3‐PDK4 regulatory axis, indicating that PDK4 is a key downstream effector molecule of HDAC3. An in vitro iron overload model was constructed to study neuronal changes, and RGFP966 at a concentration of 10 μM was chosen to intervene in neurons under iron overload (Figure 6N). The neuron's spike length was damaged after overloaded iron treatment, but this damage was reduced after inhibition of HDAC3 (Figure 6O), and the neuron's immunofluorescence intensity showed the same trend (Figure 6P). NG2 and MBP were stained to observe the number and proportion of oligodendrocyte lines and the area of single oligodendrocytes (Figure 6Q). Compared with the control group, the MBP single cell area decreased after overloaded iron stimulation, and the MBP single cell area increased after HDAC3 inhibition (Figure 6R). The proportion of OLGs increased, and the proportion of OPCs decreased (Figure 6S,T). Through both in vitro and in vivo model validations by gene overexpression, we have confirmed that HDAC3 is the core driver of ferroptosis.

## 4 Discussion

The clinical findings demonstrated oxidative stress and iron overload as key characteristics of human WMI. CPZ‐induced copper chelation disrupts the synthesis of iron–sulfur clusters in the mitochondria, leading to secondary iron overload and oxidative stress [39, 40]. Oligodendrocyte injury‐mediated myelin loss represents a hallmark pathological feature in demyelinating disorders [19]. 3D‐SEM reveals CPZ‐induced oligodendrocytopathy as progressive out‐of‐phase degeneration with ultrastructural axon‐myelin unit abnormalities during chronic progression [41]. Notably, inhibition of HDAC3 promotes OLGs maturation while inhibiting OPCs proliferation, which resolves the abnormal oligodendrocyte demyelination response. Unlike pan‐HDAC inhibitors, RGFP966's isoform selectivity minimizes off‐target effects while preserving metabolic and inflammatory balance, as evidenced by improved weight gain and motor coordination in treated mice [42]. Mechanistically, inhibition of HDAC3 enhanced oligodendroglial maturation as evidenced by increased Olig2/BrdU mature cell density concurrent with OPCs suppression, mirroring previous findings of HDAC‐mediated transcriptional regulation in MBP promoter activation [43, 44].

A clinical study revealed fetal myelin dysplasia characterized by corpus callosum thinning, diffuse white matter lesions with mineralization on MRI/neuropathology, and prominent frontal lobe iron accumulation [45]. In Jhelum's study, OLGs rapidly disappeared 2 days after the initiation of the CPZ, disappeared significantly at Week 1, and significantly increased expression of NCOA4, TFRC, and FTH was observed at Day 2 to 1 week [40], which was consistent with our research results. We chose to use RGFP966 as a protective intervention during the first week of CPZ induction in reference to this study. HDAC3 shares a highly conserved structural domain with other class I HDACs, such as HDAC1 and HDAC2. Current HDAC inhibitors typically lack selectivity for specific isoforms, thereby potentially interfering with other HDAC‐mediated physiological functions. This lack of selectivity can lead to adverse effects, including cytotoxicity and immunosuppression. To date, most approved HDAC inhibitors are multi‐targeted drugs, and many of these compounds do not effectively cross the blood–brain barrier. Treatment of white matter injury requires the use of drugs that can effectively cross the blood–brain barrier. However, most of the existing HDAC inhibitors have limited distribution in the brain due to high molecular weight or insufficient lipophilicity, which significantly affects their therapeutic efficacy. RGFP966, a selective HDAC3 inhibitor, is a promising candidate that can effectively cross the blood–brain barrier, reduce side effects, and improve efficacy. Nevertheless, further clinical studies are needed to fully elucidate its safety and efficacy. Studies have found that HDAC inhibitors have a protective effect on neurodegenerative diseases [46, 47]. HDAC3 mediates epigenetic regulation by deacetylating histones to suppress transcriptional activation [48]. Our prior investigations in lysophosphatidylcholine (LPC)‐induced focal demyelination revealed selective HDAC3 upregulation in the corpus callosum at 5 days post‐injury, unaffected in HDAC1/2/8 isoforms [28]. Extending these findings to acute whole‐brain demyelination, we observed time‐dependent HDAC3 elevation correlating with ferroptosis gene expression. Mechanistically, HDAC3 inhibition attenuates ferroptosis through two interrelated pathways. HDAC3 inhibition normalizes FTH/NCOA4‐mediated iron storage and TFRC‐dependent uptake, reducing Fe^2+^ overload and subsequent Fenton reaction. Hepcidin is a key molecule that negatively regulates iron absorption [49]. HDAC3 may contribute to intracellular iron overload by repressing the transcription of *Hamp*, which encodes hepcidin [50]. HDAC3 exacerbates neuronal oxidative damage by inhibiting the expression of superoxide dismutase (SOD2) [51]. Increased HDAC3 exacerbates lipid peroxidation through ACSL4 activation, whereas inhibition of HDAC3 reverses this effect by restoring GPX4/xCT antioxidant capacity. Iron metabolism disorder is not only a secondary phenomenon of demyelination but also a vicious cycle through the activation of ferroptosis. Iron overload drives lipid peroxidation, triggering oligodendrocyte death and subsequent iron release, which amplifies oxidative damage. This cascade disrupts myelin repair and accelerates neurological decline.

PDK4‐mediated metabolic reprogramming antagonizes ferroptosis, synergizing with xCT inhibitors in cancer models [52]. Pharmacological PDK4 inhibition activates Nrf2‐dependent antioxidant pathways and normalizes iron metabolism, whereas suppressing PDK4‐YAP/JNK signaling reduces aging phenotypes [53, 54]. PDK4 inhibition enhances mitochondrial function and redox balance, reducing oxidative stress and neurodegeneration; however, complete PDK4 ablation disrupts metabolic homeostasis [55]. Liver‐specific PDK4 knockout triggers JNK hyperactivation, GSH depletion, and hepatocyte apoptosis under stress [56], emphasizing the need for stage‐targeted therapies. In our study, at the 5th week of demyelination, lipid peroxidation in the white matter of the mouse was at its peak, and at this time, the expression of PDK4 increased significantly, which may be mediated by the increase in the level of HDAC3 protein. At the same time, the protein expression and mRNA level of PDK4 were increased after HDAC3 overexpressed virus injection, indicating that HDAC3 acts on PDK4 and increases its expression. In the early stage of demyelination, at 1 week, the level of PDK4 is low, and the binding effect of HDAC3 and PDK4 is weak at this time, and RGFP966 restores the expression of PDK4 by inhibiting HDAC3. Our study demonstrates that targeting the HDAC3‐PDK4 axis improves ferroptosis and provides a theoretical basis for the precise treatment of demyelinating diseases. This finding provides a new metabolic perspective for the regulation of ferroptosis by HDAC3.

In the present study, the role of RGFP966 in demyelination‐associated ferroptosis was elucidated by the HDAC3‐PDK4 mechanism. However, the study has some limitations. First of all, the time point focuses on the middle and late demyelination, which fails to fully reveal the dynamic changes of HDAC3. Secondly, the specific mechanism of PDK4 in ferroptosis has not been fully elucidated, and how HDAC3 precisely regulates chromatin modification of PDK4 and other ferroptosis‐related genes still needs further study. In the future, PDK4 knockout (KO)/overexpression experiments will be added and direct binding of HDAC3 and PDK4 will be verified by ChIP‐seq. In addition, to clarify the safety and effectiveness of RGFP966 in human diseases, future studies need to increase the ability of drugs to cross the blood–brain barrier. One limitation of this study is that all in vivo experiments were conducted exclusively in male C57BL/6 mice. The decision to use only male animals was based on the need to reduce biological variability, particularly given the extended experimental period of approximately 1 month. Female mice undergo estrous cycles, leading to periodic hormonal fluctuations that may significantly influence key pathophysiological processes investigated in this study, including ferroptosis susceptibility, iron metabolism, oxidative stress, and demyelination. By using male mice, we aimed to simplify the experimental design and improve the consistency and interpretability of mechanistic outcomes. However, we acknowledge that this sex‐specific approach may limit the generalizability of our findings. Emerging evidence suggests that sex hormones modulate neuroinflammatory and metabolic pathways relevant to white matter injury and ferroptosis. Therefore, future studies will incorporate both male and female animals to comprehensively evaluate potential sex differences in response to HDAC3‐targeted interventions and PDK4‐mediated iron regulation.

This study finds a novel HDAC3‐PDK4 axis that drives ferroptosis in demyelinating pathologies through epigenetic‐metabolic crosstalk. Inhibition of HDAC3, restoring iron homeostasis and reducing lipid peroxidation, is a promising strategy to halt the progression of WMI. Future work should explore combinatorial therapies targeting HDAC3 and iron chelation to synergistically enhance myelin repair in neurodegenerative disorders.

## Author Contributions

G.W., C.W., and Q.L. conceived, designed, and supervised the entire study. T.X., L.Y., W.L., F.L., T.W., and W.G. performed the experiments, acquired data, analyzed data, and drafted the manuscript. L.X. and M.F. participated in part of the experiments, acquired data, and analyzed data. G.W., T.X., and L.Y. wrote and edited the manuscript. All authors contributed to reading and approved the submitted version.

## Ethics Statement

All animal experiments were approved by the Experimental Animal Ethics Committee of Nantong University Ethics Committee (No. 2020‐39) and Care and Use Committee of Nantong University (S20221113‐903). The research was conducted in accordance with relevant ethical standards and guidelines.

## Consent

Informed consent was obtained from all individual participants included in the study.

## Conflicts of Interest

The authors declare no conflicts of interest.

## Supporting information


Data S1.

## Data Availability

The file of the unedited images is available as Data S1. Other data that support the findings of this study are available from the corresponding author upon reasonable request.
