# Supplementary material for: Targeting HDAC3 Suppresses Ferroptosis and Demyelination in White Matter Injury by Restoring PDK4‐Mediated Iron Homeostasis
Source: CNS Neurosci Ther. 2025 Jun 8;31(6):e70471. doi: 10.1111/cns.70471 (PMC12146139; doi:10.1111/cns.70471)
Supplement: Supplementary file 1 — Data S1. [file CNS-31-e70471-s001.docx]

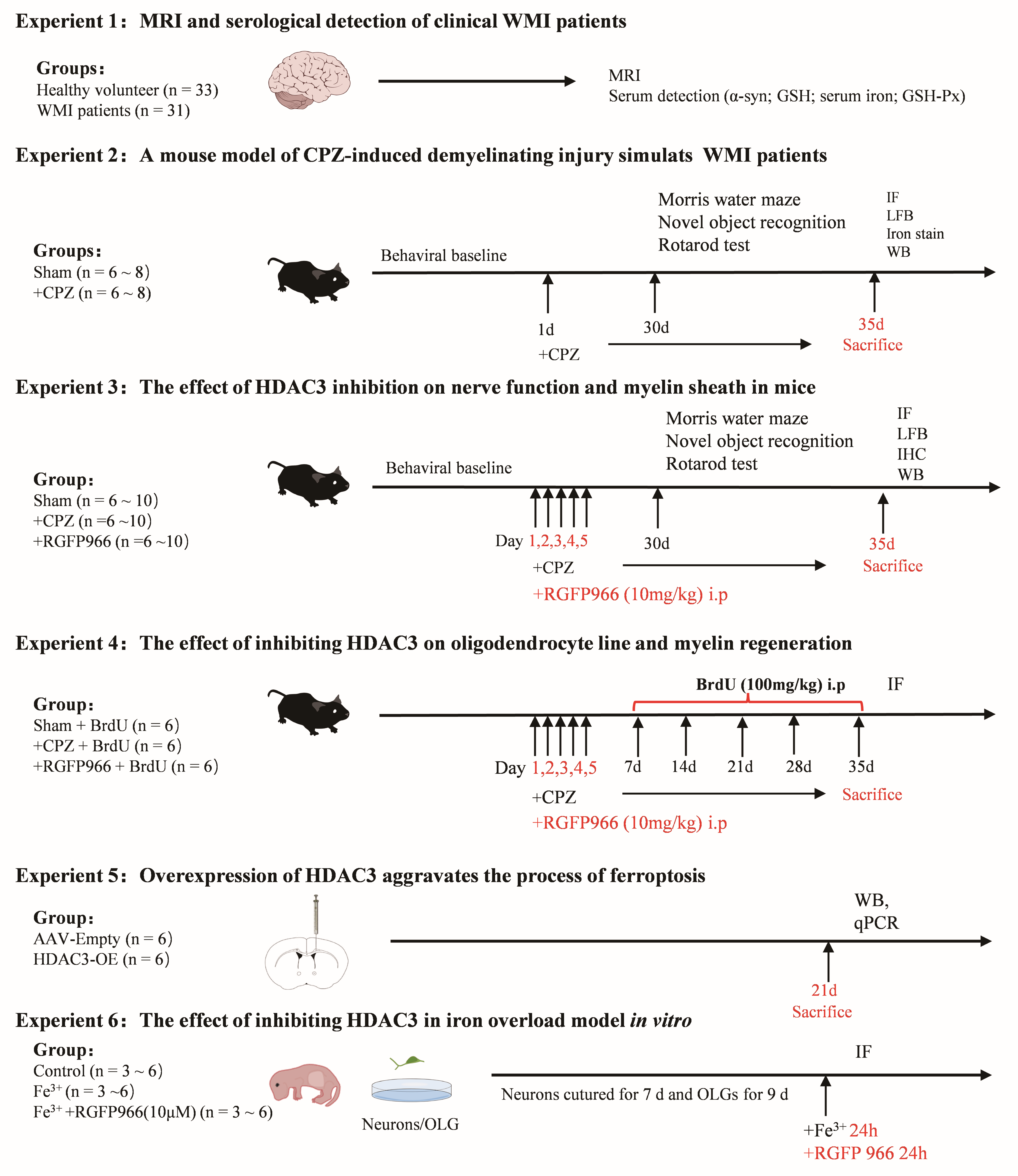


**Figure S1.** Experimental timeline

**IF:** Immunofluorescence, **LFB:** Luxol Fast Blue**, WB:** Western Blot**, HDAC3-OE:** Histone Deacetylase 3- Overexpression.

**Table S1.** List of the antibody

| **Company** | **Antibody** | **Cat.No** | **Species** | **Dilution** |
| --- | --- | --- | --- | --- |
| Novus | Ferroportin/SLC40A1 (FPN1) | NBP1-21502 R | Rabbit | 1:1000 |
| Abcam | Histone deacetylase 3 (HDAC3) | Ab137704 | Rabbit | 1:1000 |
| Abcam | APC | ab16794 | Mouse | 1:1000 |
| Thermo Fisher | Transferrin Receptor (TFRC) | 136800 | Mouse | 1:1000 |
| Abcam | Acetyl-Histone H3 | 10799 | Rabbit | 1:1000 |
| Santa-cruz | Acyl-CoA Synthetase Long Chain Family Member 4 (ACSL4) | sc-271800 | Mouse | 1:1000 |
| Abcam | Glutathione peroxidase 4 (GPX4) | ab125066 | Rabbit | 1:1000 |
| Proteintech | Solute carrier family 7 member 11 (xCT/SLC7A11) | 26864-1-AP | Rabbit | 1:1000 |
| Abcam | Ferritin light chain (Ft-L) | ab69090 | Rabbit | 1:1000 |
| Novus | Ferritin heavy chain (FTH) | NBP1-31944 | Rabbit | 1:1000 |
| Proteintech | Pyruvate dehydrogenase kinase 4 (PDK4) | 12949-1-AP | Rabbit | 1:1000 |
| Sigma | β-actin | A5316 | Mouse | 1:1000 |
| Abcam | Anti-APC | ab16794 | Rabbit | 1:1000 |
| BIOSHARP | GAM-HRP | BL001A |  | 1:10000 |
| BIOSHARP | GAR-HRP | BL003A |  | 1:10000 |

**Table S2.** List of primer sequences

| **Gene** | **Primer sequence** |
| --- | --- |
| *MLLT1* -Forward | 5'- CCAGGTGAAGTTAGAGCTGGG -3' |
| *MLLT1* -Reverse | 5'- GCTTGGGTTTAGGGAAGCTGT -3' |
| *PDK4* - Forward | 5'- CCGCTTAGTGAACACTCCTTC -3' |
| *PDK4* - Reverse | 5'- TGACCAGCGTGTCTACAAACT -3' |
| *ENPP2* - Forward | 5'- TGAGCTTCAAGAGGTTGGACC -3' |
| *ENPP2* - Reverse | 5'- CCACTTGGTAGTTGGTACAGC -3' |
| *PDK4* - Forward | 5'- CCGCTTAGTGAACACTCCTTC -3' |
| *PDK4* - Reverse | 5'- TGACCAGCGTGTCTACAAACT -3' |
| *CDH1* - Forward | 5'- CAGGTCTCCTCATGGCTTTGC -3' |
| *CDH1* - Reverse | 5'- CTTCCGAAAAGAAGGCTGTCC -3' |
| *CDO1* - Forward | 5'- GGGGACGAAGTCAACGTGG -3' |
| *CDO1* - Reverse | 5'- ACCCCAGCACAGAATCATCAG -3' |
| *HAMP* - Forward | 5'- CCTGAGCAGCACCACCTATC -3' |
| *HAMP* - Reverse | 5'-TGGGAATTGTTACAGCATTTACAGC -3' |
| *TFRC* - Forward | 5'- AATGGTTCGTACAGCAGCGGAAG -3' |
| *TFRC* - Reverse | 5'- TAGCACGGAAGTAGTCTCCACGAG-3' |
| *ACSL4* - Forward | 5'- GCGTTCCTCCAAGTAGACCAACC -3' |
| *ACSL4* - Reverse | 5'- TCAGTTCCAGCACATGAGCCAAAG -3' |
| *xCT* - Forward | 5'- TGGGTGGAACTGCTCGTAAT -3' |
| *xCT* - Reverse | 5'- AGGATGTAGCGTCCAAATGC -3' |
| *GPX4-* Forward | 5'- CCTCCCCAGTACTGCAACAG -3' |
| *GPX4-* Reverse | 5'- GGCTGAGAATTCGTGCATGG -3' |
| *FTH-* Forward | 5'- CAAGTGCGCCAGAACTACCA -3' |
| *FTH-* Reverse | 5'- ACAGATAGACGTAGGAGGCATAC -3' |
